# Alterations of white matter integrity associated with cognitive deficits in patients with glioma

**DOI:** 10.1002/brb3.1639

**Published:** 2020-05-15

**Authors:** Dongming Liu, Yong Liu, Xinhua Hu, Guanjie Hu, Kun Yang, Chaoyong Xiao, Jun Hu, Zonghong Li, Yuanjie Zou, Jiu Chen, Hongyi Liu

**Affiliations:** ^1^ Department of Neurosurgery The Affiliated Brain Hospital of Nanjing Medical University Nanjing China; ^2^ Institute of Brain Functional Imaging Nanjing Medical University Nanjing China; ^3^ Department of Radiology The Affiliated Brain Hospital of Nanjing Medical University Nanjing China; ^4^ Institute of Neuropsychiatry The Affiliated Brain Hospital of Nanjing Medical University Fourth Clinical College of Nanjing Medical University Nanjing China

**Keywords:** cognitive function, glioma, right uncinate fasciculus, structural network, white matter tracts

## Abstract

**Objective:**

This study aimed to investigate the characteristic of brain structural connections in glioma patients and further evaluate the relationship between changes in the white matter tracts and cognitive decline.

**Methods:**

This retrospective study included a total of 35 subjects with glioma and 14 demographically matched healthy controls, who underwent diffusion tensor imaging scans and formal neuropsychological assessment tests. Fractional anisotropy (FA) values of white matter tracts were derived from atlas‐based analysis to compare group differences. Furthermore, subgroup‐level analysis was performed to differentiate the effects of tumor location on white matter tracts. Partial correlation analysis was used to examine the associations between neurocognitive assessments and the integrity of tracts. Region of interest‐based network analysis was performed to validate the alteration of structural brain network in subjects with glioma.

**Results:**

Compared with controls, subjects with glioma exhibited reduced FA values in the right uncinate fasciculus. Besides, subjects with glioma exhibited worse performance in several cognitive assessments. Partial correlation analysis indicated that the FA value in the right superior longitudinal fasciculus temporal part was significantly positively correlated with scores of visual–spatial abilities in subjects with glioma in the right temporal lobe (*r* = .932, *p* = .002). Region of interest‐based network analysis revealed that subjects with glioma exhibited reduced FA, fiber length (FL), and fiber number (FN) between specific brain regions compared with controls.

**Conclusion:**

The present study demonstrated the reduced integrity of white matter tracts and altered structural connectivity in brain networks in patients with glioma. Notably, white matter tracts in the right hemisphere might be vulnerable to the effects of a frontal or temporal lesion and might be associated with deficient cognitive function.

## Graphical abstract

In this work, we explored the effect of the heterogeneous gliomas on multiple WM tracts and global structural network. We attempted to utilize tract‐based DTI analysis to identify the alteration of the white matter (WM) tracts in patients with glioma. The cognitive characteristics of the patients with glioma also studied.

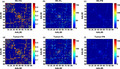

## 1 INTRODUCTION

Gliomas represent the most frequently diagnosed primary malignant intracranial neoplasms in adults, contributing to approximately 81% of all primary central nervous system tumors (Ostrom et al., 2014). Certainly, infiltrative growth associated with highly complex interactions between cortical and subcortical pathway damage is considered to be a hallmark of gliomas, and therefore, patients with glioma often present with a wide range of symptoms. Besides the most common clinical manifestations, including nausea, headache, epilepsy, and neurological deficits, an increasing number of studies have shown that patients with gliomas may suffer impairments in multiple cognitive domains (Maesawa et al., 2015; Mariano, Mazza, & Galzio, 2013; Miotto et al., 2011). Gliomas have been reported to cause various degrees of white matter fiber damage (Incekara, Satoer, Visch‐Brink, Vincent, & Smits, 2018; Soni, Mehrotra, Behari, Kumar, & Gupta, 2017). In addition, gliomas are well recognized for their infiltrative growth along the white matter tracts (Louis, 2006). Thus, a better understanding of the relationship between cognitive alterations and white matter integrity in patients with glioma could potentially optimize the preoperative evaluation and the rehabilitation strategies, thereby improving patients' quality of life.

Diffusion tensor imaging (DTI) is a widely used MRI technique that can noninvasively reveal the diffusion of water molecules within the brain tissue to detect, visualize, and quantify the integrity of brain microstructure (Carlo, Jezzard, Basser, Barnett, & Di Chiro, 1996). Parametric quantitative information on white matter (WM) tract integrity can be derived from tractography and reflected as a value of fractional anisotropy (FA). Therefore, FA can measure changes in water diffusion and provide complementary information about the white matter microstructure, especially the development of white matter fiber tracts (Alexander et al., 2011). FA is commonly referred to as a summary measure of microstructural integrity of WM tracts and is a normalized standard deviation of the eigenvalue between 0 and 1. A reduced FA in a WM tract usually indicates a change in the integrity of the fiber bundle (Alexander et al., 2011; Incekara et al., 2018). Diffusion tensor tomography (DTT) evaluates water diffusion in the WM of the brain and shows subcortical tract integrity based on indices such as FA, mean diffusivity, and radial diffusivity. DTT allows visualization of the exact location of brain tumors that may be relevant to specific WM tracts and has been widely used (Yu, KunCheng, Yun, XunMing, & Wen, 2005). Previous studies have shown that gliomas may cause alterations in WM tract integrity to vary degrees due to their different locations and infiltrative growth (Incekara et al., 2018; Ilhami et al., 2011; Soni et al., 2017). As an intra‐axial primary brain tumor, malignant glioma cells exhibit preferential invasion along WM tracts in the brain tissue (Bellail, Hunter, Brat, Tan, & Van Meir, 2004; Louis, 2006; Mandonnet, Capelle, & Duffau, 2006). Due to underlying neoplasm invasiveness and migration along WM fibers (Bellail et al., 2004; Rao et al., 2013), in addition to the specific WM tracts that are directly affected by tumors, it is also interesting to study distal WM tracts that may be potentially affected by the invasive tumors.

Previous studies have suggested that the brain structural network of patients with brain disorders (including stroke, cerebrovascular disease, and tumor‐related lesions) may be disrupted, some of which are related to cognitive dysfunction (Du et al., 2019; Ille, Engel, Kelm, Meyer, & Krieg, 2018; Urbanski et al., 2011). Despite a growing body of the literature on examining tumor‐affected brains through DTT technique (Abhinav, Yeh, Mansouri, Zadeh, & Fernandez‐Miranda, 2015; Conti Nibali et al., 2019; Incekara et al., 2018), a few studies have endeavored to study the effects of glioma from a whole‐brain perspective. Nakajima et al. demonstrated that high‐level mentalizing processing was associated with structural connectivity networks, which were influenced by the effect of right cerebral hemispheric glioma and its resection (Nakajima et al., 2018). Zhou et al. reported altered connection density and brain anatomical networks in patients with a brain tumor (Zhou et al., 2016). Excessive attention to a specific WM tract may lead us to ignore the effect of tumors on distant tracts and whole‐brain structural networks. However, to understand the putative long‐range effects of glioma may allow more accurate prediction of the effects of glioma resection and insight into mechanisms of subcortical structural network. Moreover, such research may further contribute to the understanding of the clinical–psychological symptoms in patients with glioma.

In the present study, we acquired DTI data of 35 glioma patients to study the effect of the gliomas on multiple WM tracts and structural networks. In addition, we sought to clarify the association between changes in structural integrity and impairment to certain cognitive domains. We hypothesized that it is not only the subcortical structures subjected to anatomical damage that exhibits impairments, but also the interconnected remote regions that seem to be affected by the lesions (Duffau, 2014). Tumor damage can affect the inherent brain structural connectome and cause network dysfunction (Sharp, Scott, & Leech, 2014).

## 2 METHODS

### 2.1 Study participants

This retrospective study included a total of 53 patients with histopathologically (postoperatively) confirmed malignant primary glioma at the Department of Neurosurgery of the Affiliated Brain Hospital of Nanjing Medical University, Jiangsu Province, China. Patient inclusion criteria were as follows: (1) histopathologically confirmed malignant primary glioma according to the World Health Organization (WHO) classification; (2) no previous history of craniocerebral surgery, head trauma, psychological disease, or cerebrovascular disease or no history of substance abuse, including tobacco, alcohol, or other psychoactive substances; (3) no history of cerebral radiotherapy or temozolomide chemotherapy; and (4) no or slight neurological focal deficit including aphasia or paresis. Patients with recurrent glioma (*n* = 2), marked peritumoral edema associated with midline shift (*n* = 7), or without sufficient available DTI data (*n* = 9) were excluded from the study. A total of 35 subjects (19 males and 16 females, with an average age of 49.60 years) met the inclusion and exclusion criteria and were included for the data analysis (the demographic characteristics are summarized in the supplementary materials, Table S1). For comparison, fourteen demographically matched (sex, age, and education) healthy controls (HCs) were also recruited from the local community between 2013 and 2015. To ensure these individuals represented a healthy comparison group, HCs were evaluated using unstructured clinical interviews to exclude individuals who had a history of severe systemic disease, head trauma, or psychological disorders. All subjects were native Chinese descent and right‐handed according to the Edinburgh Handedness Inventory.

Of the 35 subjects with glioma, according to WHO classification criteria, 16 subjects exhibited low‐grade glioma (LGG) (WHO I or II), and 19 subjects exhibited high‐grade glioma (HGG) (WHO III or IV). Besides, 15 subjects had glioma located in the left hemisphere and 20 had in the right hemisphere. The histopathological type of the tumors and the WHO grade of the gliomas were determined by a neuropathologist from tumor tissue obtained during the surgical resection. Localization of the tumor was determined by a neuroradiologist and a neurosurgeon using preoperative T1‐, T2‐weighted and T1‐contrasted images. The subjects' clinicopathological characteristics are listed in Table 1. Written informed consent was obtained from all participants. This study was approved by the Institutional Ethical Committee for Clinical Research of the Affiliated Brain Hospital of Nanjing Medical University.

**TABLE 1:** Demographic characteristics of patients with gliomas

Characteristic	Value/number
Gender (male/female)	19/16
Mean age in years (*SD*)	49.6 (14.3)
Tumor grade (WHO grade)	
LGG (Ⅰ–Ⅱ)	16
HGG (Ⅲ–Ⅳ)	19
Histopathological subtype (WHO grade)	
Astrocytoma (Ⅰ–Ⅱ)	11 (31.4%)
Ganglioglioma (Ⅰ–Ⅱ)	3 (8.6%)
Oligodendroglioma (Ⅱ)	2 (5.7%)
Anaplastic astrocytoma (Ⅲ)	3 (8.6%)
Anaplastic oligodendroglioma (Ⅲ)	5 (14.3%)
Glioblastoma (Ⅳ)	11 (31.4%)
Tumor location	
Left	15 (42.9%)
Frontal	6 (17.1%)
Temporal	6 (17.1%)
Parietal	2 (5.7%)
Occipital	1 (2.9%)
Right	20 (57.1%)
Frontal	6 (17.1%)
Temporal	11 (31.4%)
Parietal	3 (8.6%)
Occipital	0 (0%)

### 2.2 MRI data acquisition

The subjects included in this retrospective study were scanned between 2013 and 2015 for presurgical planning. All MRI images were acquired in 3.0 Tesla Verio Siemens scanner (Siemens) in the Department of Radiology, the Affiliated Brain Hospital of Nanjing Medical University. The structural scans were axially acquired using three‐dimensional (3D) T1‐weighted magnetization‐prepared rapid gradient echo (MPRAGE) with the following acquisition parameters: repeat time (TR) = 1,900 ms; echo time (TE) = 2.49 ms; time inversion (TI) = 900 ms; matrix = 256 × 256; flip angle = 90°; slice thickness = 1 mm; slice gap = 0.5 mm; and slice number = 176. Diffusion MRI data were carried out using an echo planar imaging (EPI) sequence with the following parameters for three times: diffusion encoding in 32 independent, noncollinear directions with a *b*‐value = 1,000 s/mm^2^ and one additional image with no diffusion weighting (*b* = 0); slice number = 62; TR = 6,500 ms; TE = 95 ms; slice gap = 3 mm; slice thickness = 3 mm; flip angle = 90°; field of view (FOV) = 120 mm × 120 mm; and acquisition matrix = 128 × 128.

### 2.3 Data preprocessing

The DTI data were preprocessed and analyzed by PANDA (Pipeline for Analyzing Brain Diffusion Images) software (http://www.nitrc.org/projects/panda) (Cui, Zhong, Xu, He, & Gong, 2013). The preprocessing steps were performed as described previously (Bai et al., 2012; Wang et al., 2016). Briefly, (a) the DICOM files of all subjects were converted into NIfTI images using the dcm2nii tool embedded in MRIcron; (b) the extraction of brain tissue and structure, this step also yielded the brain mask for the subsequent processing steps; (c) realignment; (d) each diffusion‐weighted image was coregistered to the b0 image using affine transformation to correct the head motion artifacts and distortions caused by eddy currents, and the diffusion gradient directions were also adjusted accordingly; (e) calculation of fractional anisotropy (FA); (f) reconstruction of diffusion tensor tractography; and (g) tractography and network construction to produce 3D streamlines representing fiber tract connectivity (Mori, Crain, Chacko, & van Zijl, 1999).

#### 2.3.1 Atlas‐based analysis of DTI values

The atlas‐based analysis was used to analyze the differences between glioma patients and HCs and subgroup levels (12 patients with frontal glioma and 11 right temporal lobe glioma). However, due to data limitations, the changes in DTI values in patients with glioma in the left temporal, the parietal, and the occipital lobe could not be investigated. Briefly, we defined WM tracts into 20 tracts using the JHU WM tractography atlas (http://cmrm.med.jhmi.edu/) (the defined WM tracts and abbreviation are listed in Table S2). The WM atlas in the standard space allows for parcellation of the entire brain WM into 20 regions of interests (ROIs), each representing a labeled region in the atlas. Subsequently, the 20 tracts were used as masks to extract the mean diffusion measures within the regions. The regional diffusion metrics (such as FA) were generated from PANDA by averaging the values within the specific region of each WM atlas. Atlas‐based values can be further exported to Statistical Package for the Social Sciences (SPSS) for statistical analysis (Cui et al., 2013). The significantly altered FA of WM tracts was selected and extracted by the tract‐based masks for the further correlation analysis.

#### 2.3.2 White matter network construction

##### Definition of network nodes

We used an automated anatomical labeling (AAL) template (Tzourio‐Mazoyer et al., 2002), an extensively used high‐resolution T1‐weighted brain parcellation, to parcel the brain into 90 cortical and subcortical ROIs (45 in each hemisphere without cerebellar regions). The names and abbreviations of these ROIs are summarized in supplementary materials, Table S3. These 90 ROIs were used as nodes for global network connectivity analysis.

##### Constructing networks using deterministic tractography

ROI‐based whole‐brain deterministic tractography analysis was generated using PANDA, and the streamlines would be terminated unless the voxel with an FA < 0.2 or met a fiber with turning angle >45°. For each pair of above‐mentioned network nodes, fibers with two terminal points located in their respective regions were considered connected to the two nodes. Based on the linking fibers, three basic weighted matrices, FA‐weighted matrix, length‐weighted matrix, and number‐weighted matrix, were calculated using PANDA, which resulted in three weighted matrix maps in HCs and subjects with glioma, respectively. In a resultant matrix, each column or row represents a node or brain region defined by the AAL template. Subsequently, individual‐level white matter connectivity network was constructed based on the value of FA, fiber length (FL), and fiber number (FN), respectively (Bullmore & Sporns, 2009; Cui et al., 2013; Edward & Bassett, 2011).

### 2.4 Neuropsychological assessment

All participants included in the present study underwent an unstructured clinical interview. Several classical neurocognitive assessments were performed for each participant, including a block design (Wechsler Adult Intelligence Scale‐Chinese Revision; WAIS‐RC) digit span test (DST) (Dai, Ryan, Paolo, & Harrington, 1990), digital symbol substitution test (DSST) (Tang, Lau, & Chang, 1996), mapping test (Ryan, Dal, Paolo, & Harrington, 1992), similarity test (Ward & Ryan, 1997), and visuospatial test (Ryan et al., 1992). DST is a standard test of a person's working memory for numbers to evaluate attention and temporary working memory abilities. DSST is a test to evaluate the information processing speed and executive abilities of the subjects by asking them to convert the numbers into corresponding symbols. In addition, mapping is used to assess the subjects' executive, visual recognition, and visual understanding abilities by filling in incomplete geometric figures. Similarity is a semantic test to evaluate logical thinking and perceptual speed ability by classifying the given words. Visuospatial test is a building block pattern recognition test to estimate the visual and spatial recognition abilities of the subjects. Moreover, memory test (Wechsler Memory Scale‐Chinese Revision; WMS‐RC) was also performed to estimate memory functions of the subjects (Kent, 2013). The results were Z‐transformed to facilitate further partial correlation analysis.

### 2.5 Statistical analysis

Statistical analyses were performed with SPSS software version 19.0 (IBM, Armonk, New York). The two‐tailed two‐sample *t*‐tests and chi‐square tests were performed to compare demographic, atlas‐based FA values and neurocognitive variables between HCs and patients with glioma and/ or subgroups. Analysis of covariance (ANCOVA) was performed to compare the mean FA values of the 20 WM tracts between patients with glioma LGGs (WHO I–II) and HGGs (WHO III–IV). We also carried ANCOVA using groupings based on WHO II–III versus WHO IV gliomas. Clinical variables including age, gender, and education were treated as covariates with no interest in the comparison model. Partial correlation analysis was performed to assess the associations between WM tract integrity (i.e., FA values) and neurocognitive scores. Gender, age, and education were considered as covariates. Differences were considered statistically significant at a *p*‐value of <.05 (two‐tailed). The FA, FL, and FN connectivity matrices at the group level were calculated and analyzed using the PANDA software package. The two‐sample *t*‐tests were conducted to compare the FA, FL, and FN connectivity matrices. A *p*‐value of <.05 for FA (false discovery rates (FDRs) corrected) was considered statistically significant. The presentations of the significant differences for regional diffusion metrics (such as FA, FL, and FN) between the two groups were generated using BrainNet Viewer (http://www.nitrc.org/projects/bnv) (Figure 5).

## 3 RESULTS

### 3.1 Demographic and neurocognitive characteristics

**FIGURE 1:**
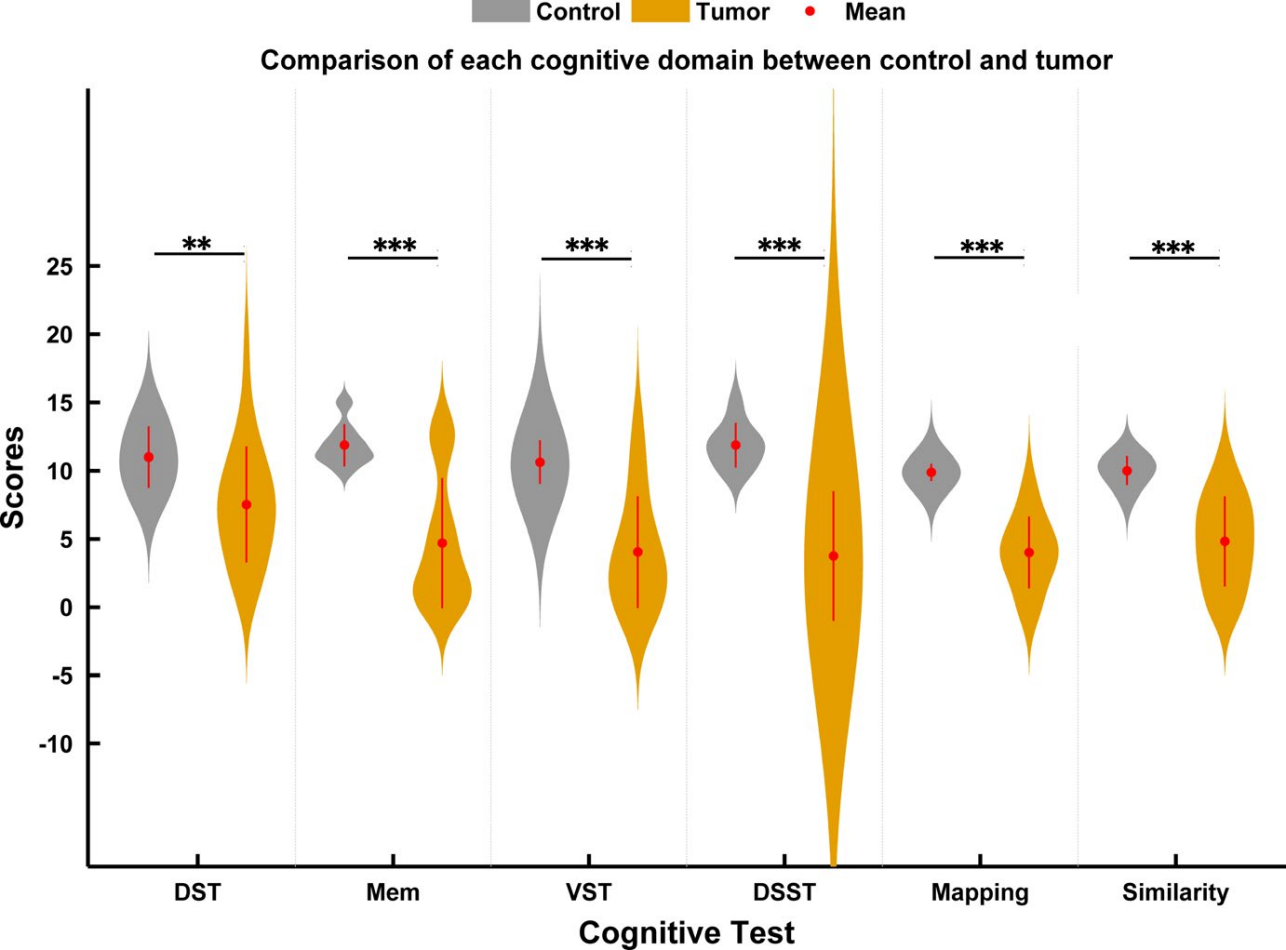
Comparison of the cognitive domain between HCs and all patients with glioma. DSST, digital symbol substitution test; DST, digit span test; Mem, memory test; VST, visuospatial test. Notes: **p < .01, ***p < .001

The demographic characteristics of patients with glioma and HCs are presented in Table 2. No differences were observed for age, gender, or education between HCs and patient groups or subgroups (*p* > .05). Eight participants with glioma failed to finish all cognitive tests. Subjects with glioma performed worse than HCs in all six cognitive tests (DST, DSST, memory, visuospatial, mapping, and similarity, all *p* < .001 except DST *p* < .0037) (Figure 1).

**TABLE 2:** Demographics and cognitive measures of patients with glioma and healthy controls (HCs)

Items	Controls (*n* = 14)	Glioma (*n* = 35)	*T* value (χ^2^)	*p* ^1^	*p* ^2^	*p* ^3^
Age (years)	48.57 (8.7)	49.60 (14.3)	−0.308	.760	.587	.693
Gender (male/female)	6/8	19/16	0.523	.470	.462	.165
Education level (years)	9.50 (4.8)	8.20 (3.6)	1.037	.305	.570	.635
Scores of each cognitive domain						
DST	11.00 (2.3)	8.23(4.5)	2.204	.0037^**^	.292	.102
Memory test	11.88 (1.6)	4.91 (4.6)	6.233	<.001^***^	<.001^***^	.022^*^
Visuospatial test^a^	10.63 (1.6)	4.18 (4.2)	6.081	<.001^***^	<.001^***^	<.001^***^
DSST	11.88 (1.6)	5.00 (5.1)	5.651	<.001^***^	.029^*^	.006^**^
Mapping	9.88 (0.6)	4.27 (2.8)	8.780	<.001^***^	<.001^***^	.004^**^
Similarity	10.0 (1.1)	4.64 (3.9)	5.875	<.001^***^	.006^**^	.017^*^

Note

Values are expressed as the mean (standard deviation; *SD*). *t* column is the values of two‐sample *t*‐test between all patients with glioma and HCs. All *p‐*values were obtained using *t*‐test except for gender (chi‐square test). *p* ^1^ representing the comparison between HCs and all patients with glioma. *p* ^2^ representing the comparison between HCs and patients with glioma located in the frontal lobe. *p* ^3^ representing the comparison between HCs and patients with glioma located in the right temporal lobe. ^*^Significant differences were observed between HCs and patients with glioma or patients' subgroup. ^*^ *p* < .05, ^**^ *p* < .01, ^***^ *p* < .001. There were 8 patients who did not complete all cognitive tests.

Abbreviations: DSST, digital symbol substitution test; DST, digit span test.

^a^ Four patients with right temporal lobe glioma did not complete this test.

### 3.2 Atlas‐based analysis of FA values

**FIGURE 2:**
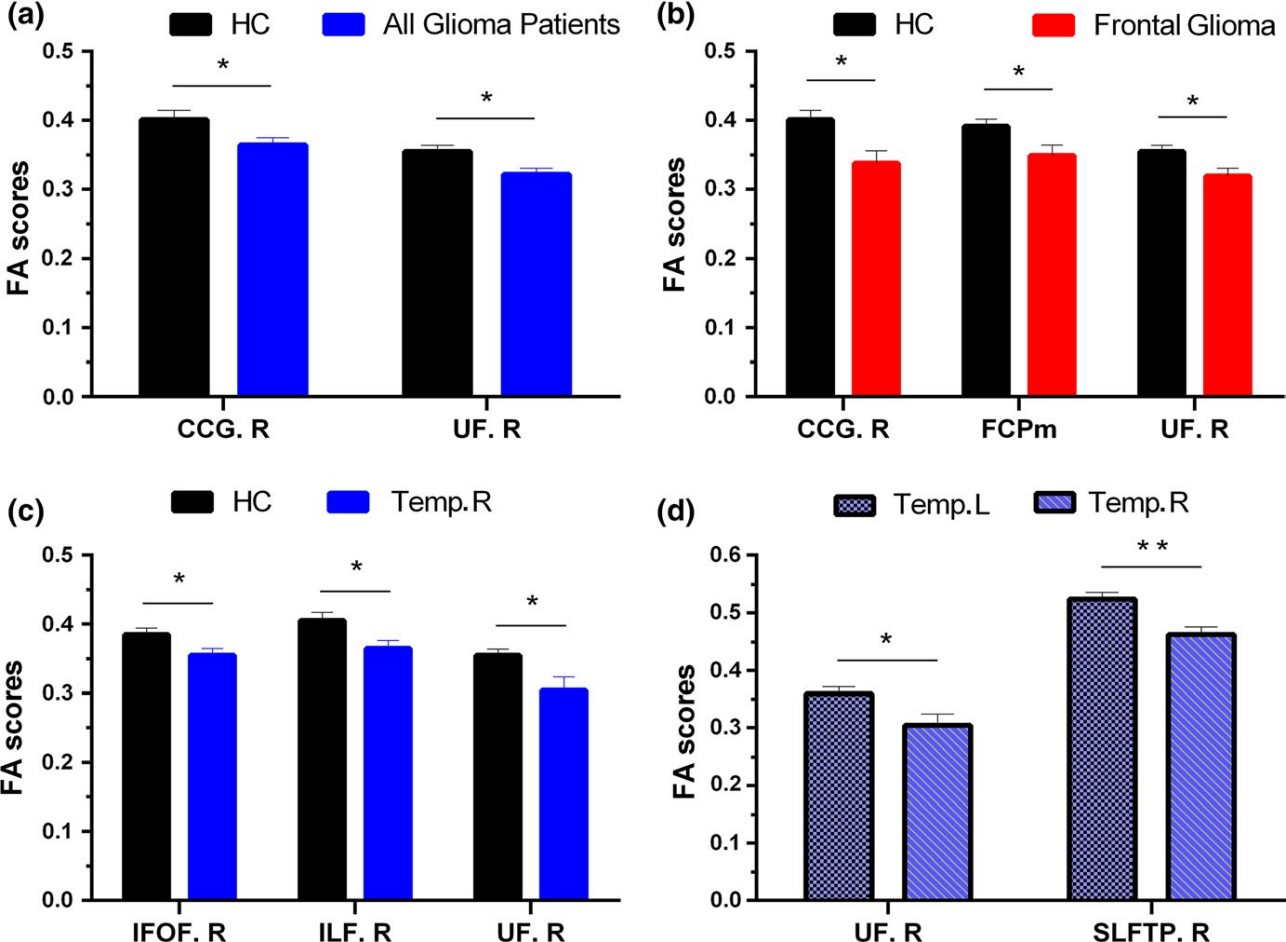
Comparisons of fractional anisotropy (FA) between HCs and all patients with glioma (a), HCs and patients with glioma located in the frontal lobe (b), HCs and patients with glioma located in the right temporal lobe (c), and patients with glioma located in the left temporal lobe and patients with glioma located in the right temporal lobe (d). Notes: Data are expressed as the mean FA value (error bars representing standard error of the mean). *p < .05, **p < .01. No significance was found between HCs and patients with gliomas located in the left temporal lobe. CCG.R, right cingulum (cingulate gyrus); FCPm, forceps minor; IFOF.R, right inferior fronto‐occipital fasciculus; ILF.R, right inferior longitudinal fasciculus; SLFTP.R, right superior longitudinal fasciculus temporal part; Temp.L/R, patients with glioma located in the left/right temporal lobe; UF.R, right uncinate fasciculus

Compared with HCs, subjects with glioma had significantly lower values of FA in two WM regions, including the right cingulate gyrus (CCG.R, *p* = .048, *t *= −2.034) and the right uncinate fasciculus (UF.R, *p* = .013, *t *= −2.593) (Figure 2a). However, using tumor grade‐based grouping, two different analyses (ANCOVA) found no significant difference in their effect on FA values.

### 3.3 Atlas‐based analysis at subgroup level

According to different anatomical locations of brain tumors, subjects with gliomas were divided into two subgroups. The two subgroups include frontal lobe (*n* = 12) and right temporal lobe gliomas (*n* = 11), representing approximately 34.3% and 31.4% of the total patients, respectively. As presented in Figure 2b, subjects with glioma in frontal lobe exhibited lower mean FA in CCG.R (*p* = .011, *t* = −2.775), UF.R (*p* = .022, *t* = −2.456), and forceps minor (FCPm, *p* = .029, *t* = −2.321) compared with that in HCs. Furthermore, in addition to lower mean UF.R (*p* = .019, *t* = −2.522), subjects with glioma in right temporal lobe also exhibited restrained FA in right inferior fronto‐occipital fasciculus (IFOF.R, *p* = .048, *t *= −2.090) and right inferior longitudinal fasciculus (ILF.R, *p* = .026, *t *= −2.374) (Figure 2c). Furthermore, the subgroup of subjects with glioma located in right temporal lobe showed lower FA in UF.R (*p* = .033, *t *= −2.355) and right SLF temporal part (SLFTP.R *p* = .009, *t *= −3.010) compared with subjects with glioma in the left temporal lobe (Figure 2d).

### 3.4 Correlation between WM tracts and neurocognitive assessment measures

**FIGURE 3:**
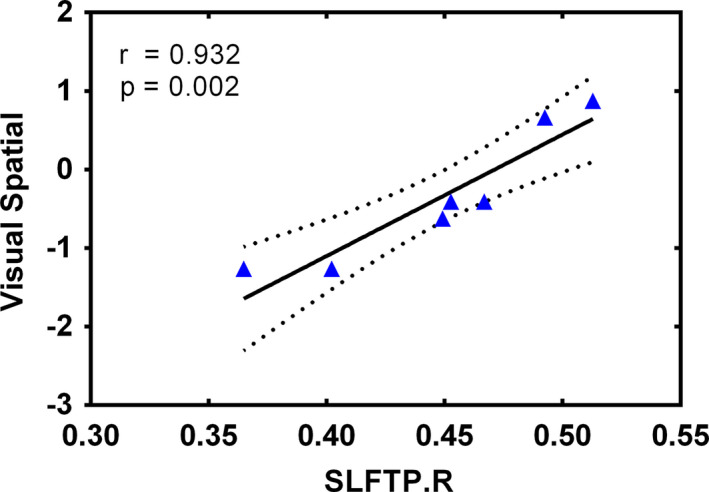
Association of significantly altered FA values of SLFTP.R and declined visual–spatial measures. Notes: ▲ represents values of patients with glioma located in the right temporal lobe. Four patients did not complete all cognitive tests. The solid line and dashed lines represent the best‐fit line and 95% confidence interval of partial correlation, respectively. SLFTP.R, right superior longitudinal fasciculus temporal part.

As illustrated in Figure 3, among subjects with glioma located in the right temporal lobe group, visual–spatial scores were positively correlated with the FA of SLFTP.R (*r* = .932, *p* = .002). No significant associations between the decreased FA of WM tracts and altered cognitive functions were observed in subjects with frontal glioma.

### 3.5 Characteristics of the structural network in patients with glioma

**FIGURE 4:**
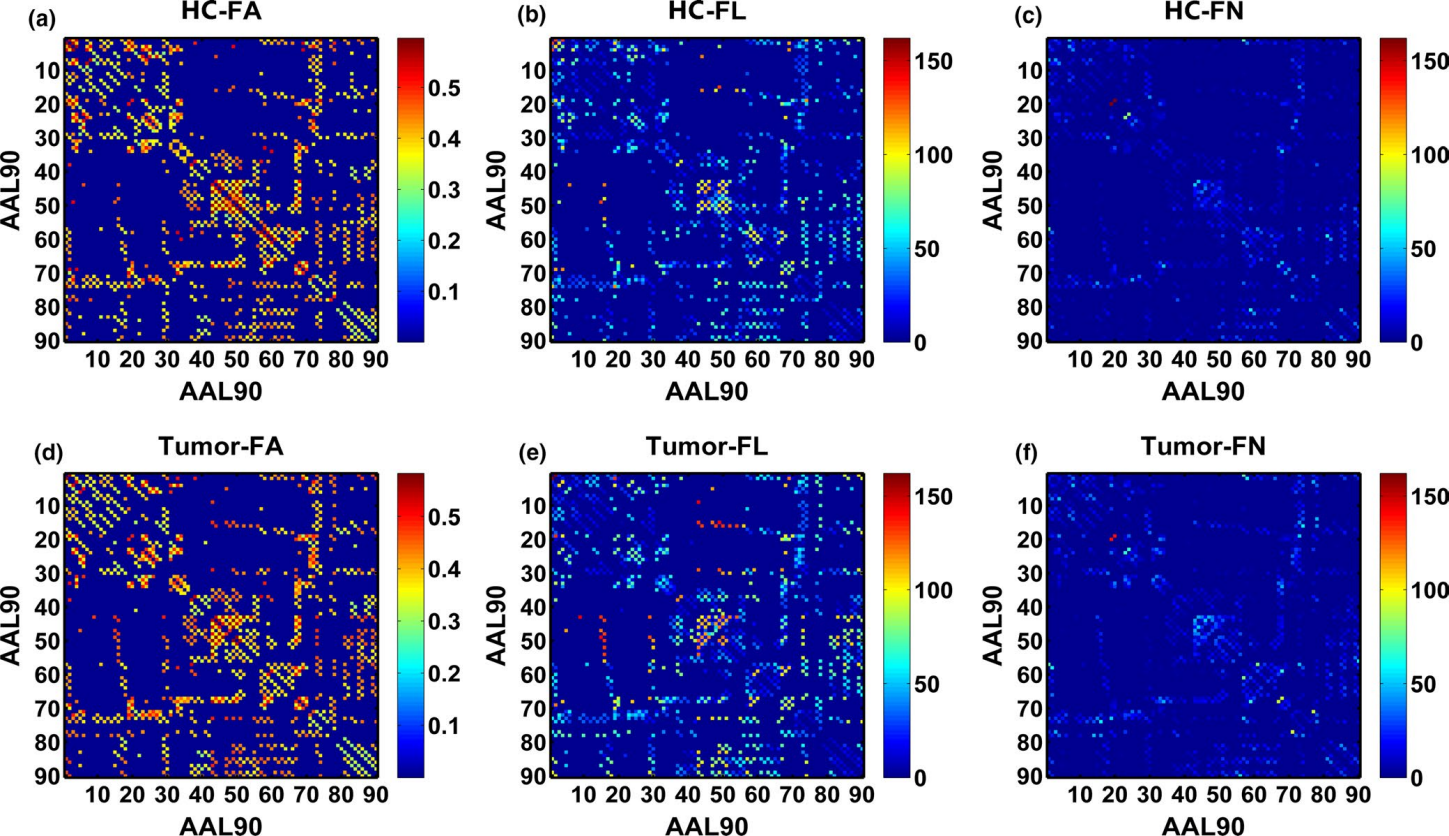
The resultant structural network matrices weighted by averaged fractional anisotropy (FA), fiber length (FL), and fiber number (FN) in HCs (a‐c) in patients with glioma (d‐f), respectively. Each 90 × 90 connectivity matrix represents the structural network of the whole cerebrum weighted by averaged FA, FL, and FN, respectively. Each column or row of the matrix represents a cortical region of the AAL template. Figure a‐c is FA‐, FL‐, and FN‐weighted network in the HCs and Figure c‐f in patients with glioma.

**FIGURE 5:**
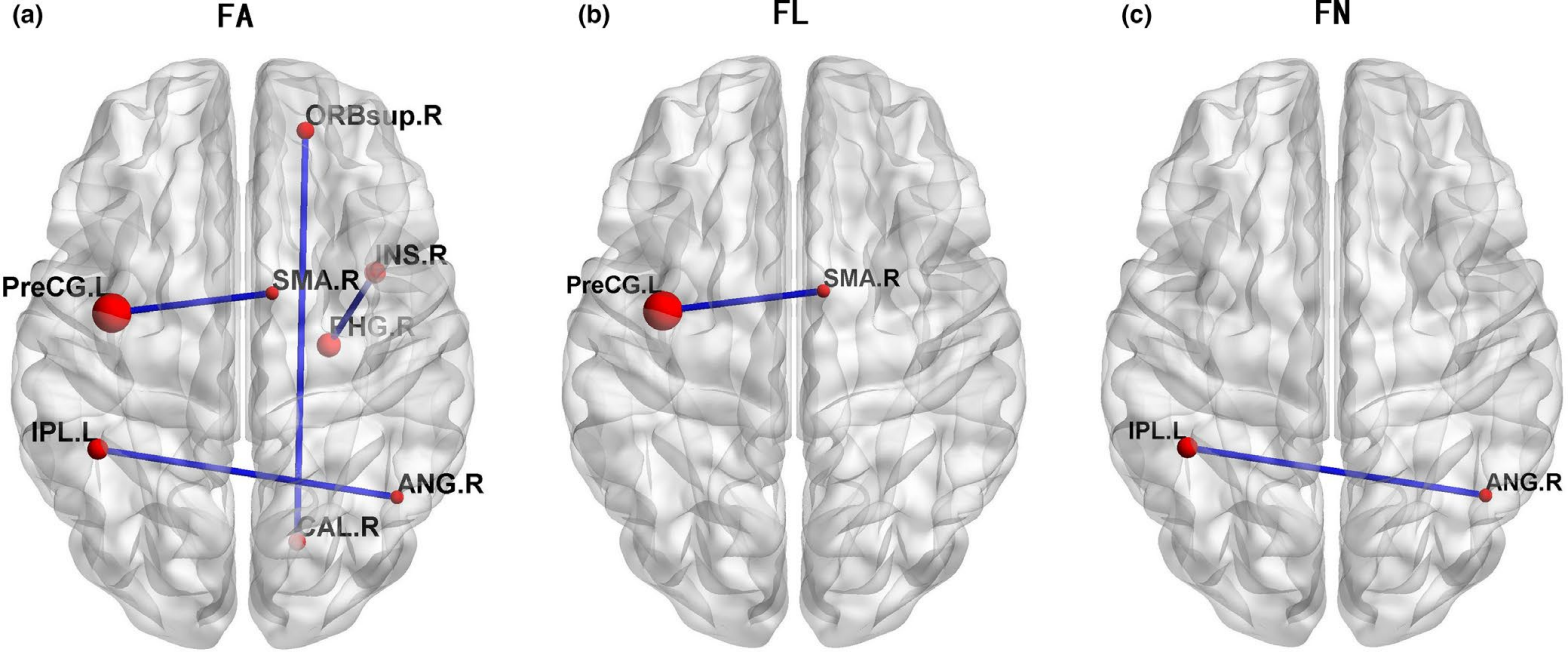
Each red node represents a cortical region of the AAL template. The edges between two seeds were weighted by averaged FA, FL, or FN value. Compared with HCs, the significantly reduced value of fractional anisotropy (FA) (*p* < .05 FDR corrected), fiber length (FL) (*p* < .001), and fiber number (FN) (*p* < .001) in patients with glioma was presented in Figure a‐c, respectively.

Successful tractography was performed for both subjects with glioma and HCs. Figure 4 presents three resultant network matrices weighted by averaged FA, FL, and FN at the group level, respectively. Significant differences in FA, FL, and FN between the two groups are presented in Figure 5. For areas of differences in average FA values between subjects with glioma and HCs, two‐sample *t*‐test revealed four significantly reduced FA values between eight specific brain regions, including the FA between left precentral gyrus (PreCG.L) and right supplementary motor area (SMA.R), the FA between left inferior parietal lobule (IPL.L) and right angular gyrus (ANG.R), the FA between right superior orbitofrontal cortex (ORBsup.R) and right calcarine (CAL.R), and the FA between right parahippocampal gyrus (PHG.R) and right insula (INS.R) (all *p* < .05, FDR corrected). For areas of significant differences in fiber length, the subjects with glioma exhibited significantly lower average FL between PreCG.L and SMA.R as compared with HCs (*p* < .001). Furthermore, compared with HCs, a significant decline in FN between IPL.L and ANG.R was also detected in patients with glioma (*p* < .001).

## 4 DISCUSSION

The present study identified that patients with glioma exhibited deficits in all of the cognitive domains assessed in our study (including DST, memory, visual–spatial, DSST, mapping, and similarity). Furthermore, patients with glioma exhibited altered FA in certain specific WM tracts (such as UF.R) as compared with HCs. Notably, a significant correlation was found between the FA in SLFTP.R and the scores of visual–spatial assessments. In a whole‐brain structural network, subdued FA, FL, and FN were observed between certain brain regions in the patients with glioma. Taken together, these results suggest that patients with glioma may exhibit microstructural changes in white matter, leading to the abnormalities in the structural connectivity network, which might explain the widespread cognitive dysfunction in patients with glioma (Korgaonkar, Fornito, Williams, & Grieve, 2014; Zhou et al., 2016).

### 4.1 DTI measures of WM tracts

From the atlas‐based analysis, a reduced FA of CCG.R and UF.R in subjects with glioma was detected. It seems that gliomas engendered worse consequences on the fibers located in the right hemisphere (such as UF.R and CCG.R). However, since the exact location of gliomas may have a direct impact on the specific fiber bundles, analysis at the subgroup level is important. Within the subset of 12 subjects with glioma located in the frontal lobe, in addition to UF.R and CCG.R, the reduced value of FA was also identified in FCPm. Edematous changes in the infiltrative tumor in WM tracts may induce a reduction in FA (Kinoshita, Nakada, Okita, Hamada, & Hayashi, 2014), which may result in the lower FA value in FCPm in patients with frontal glioma. Considering the anatomical position of those fibers, we speculated that the direct impacts of the frontal neoplasms were presented on the adjacent WM tracts. Therefore, it was not surprising to notice reduced FA values in IFOF.R, ILF.R, and UF.R in 11 subjects with glioma in the right temporal lobe. The decreased FA of UF.R persisted when the comparison between subjects with glioma located in the left and right temporal lobes was made. We further performed the process again by controlling for the tumor side (left *n* = 15; right *n* = 20). Reduced FA of CCG.R and UF.R in subjects with right hemispheric glioma could still be detected. Considering that frontal lobe gliomas can be further divided into left and right lobes, we also did a subgroup analysis of all subjects with frontal glioma (the details and results are listed in supplementary materials, Results S3.3). This kind of effect on the integrity of UF.R still exists when considering the sides of the frontal gliomas. These results indicated that the integrity of particular fibers (such as UF.R) may be more sensitive to the presence of glioma located in the frontal lobe and right hemisphere. In line with this view is evidence that microstructural WM alterations of UF.R can also be detected in patients with craniopharyngioma (Fjalldal et al., 2018). Besides, Highley et al. have indicated that the uncinate fasciculus in the right hemisphere is larger and comprised of more fibers than in the left hemisphere of healthy individuals. Our findings may be attributed to the asymmetry of UF.R observed in the healthy brain (Highley, Walker, Esiri, Crow, & Harrison, 2002; Shu, Liu, Duan, & Li, 2015).

In addition, for subjects with different pathological types of gliomas, ANCOVA yielded no significant difference in FA values of WM tracts among subjects with LGG and HGG, nor was it after using another classification method (WHO II–III and WHO IV). Similarly, Incekara et al. found no significant difference in FA values of the 3 linguistic WM tracts between LGG and HGG groups. This may be possibly ascribed to the fact that FA is not very specific to the alteration types in WM tracts (Alexander et al., 2011). As a rule, HGG is more infiltrative and destructive than LGG. Whether and how HGG affects FA and/or other DTI values (such as radial diffusivity or apparent diffusion coefficient) in peritumoral WM tracts differs from LGG. There is still some controversy (Lu et al., 2004; Miloushev, Chow, & Filippi, 2015; Smitha, Gupta, & Jayasree, 2013). This could be explained by the systematic factors or differences in methodology and the type of datasets used. Heterogeneity of tumor distribution and the small sample size could also have minimized the difference between LGG and HGG in our study. Therefore, additional studies with larger sample size and precise subgrouping are needed for further validation.

### 4.2 Association between alterations in the FA and impaired cognitive abilities

In the present study, subjects with glioma exhibited worse performance in several cognitive domains (including executive and memory functions), which is consistent with previous studies (Mariano et al., 2013; Satoer et al., 2014). Furthermore, studies have also reported the possible relationship between altered WM tracts and cognitive impairments in patients with brain tumors. Incekara et al. indicated that language and attention deficits were significantly associated with altered FA values of several WM tracts in patients with glioma (Incekara et al., 2018). In our study, the only cognitive assessment associated with altered FA was the visual–spatial test, which has been reported to be associated with the FA values of the posterior cingulum and right frontal–parietal regions (Kantarci et al., 2011; Mabbott, Noseworthy, Bouffet, Laughlin, & Rockel, 2006). Our results revealed that among patients with glioma located in the right temporal lobe group, the reduced FA of SLFTP.R was significantly positively associated with the visual–spatial scores. Although several variations exist, we speculate that local invasive gliomas might affect cognitive function by disrupting the integrity of WM tracts and these impairments may have a wide range of effects on multiple cognitive functions.

### 4.3 Alteration of the structural network in patients with glioma

Although more and more literature indicates that the brain functional network of glioma patients has changed, there are still few studies dedicated to exploring the structural network of a brain with glioma (Ghinda, Wu, Duncan, & Northoff, 2018). Zhou et al. revealed the disturbing connection density and altered brain anatomical networks in patients with glioma (Zhou et al., 2016). Besides, Bahrami et al. also reported altered network topology in patients with primary brain tumors following fractionated radiotherapy, which might contribute to the delayed cognitive impairments observed in patients with brain tumors (Bahrami et al., 2017).

The structural network of glioma patients changes in the form of altered FA, FL, and FN values between the regions located within the hemisphere (the FA between CAL.R and ORBsup.R) and across the hemisphere (the FA and FN between LPL.L and ANG.R) (Figure 5). Irrespective of the side of the lesions, the impairment of the cross‐hemispheric FA, FL, and FN indicated that the structural connections between the bilateral hemispheres might be affected by the gliomas. Previous functional MRI studies have also confirmed that patients with glioma may experience dysfunctions of cross‐hemispheric resting‐state networks (e.g., the default mode network, DMN) (Maesawa et al., 2015; Zhang et al., 2016). Conceivably, the functional aberrations might be attributed to the impaired integrity of the interhemispheric structural connections. Interestingly, left inferior parietal lobule (IPL.L) and right angular gyrus (ANG.R) are considered the classic brain regions in the DMN (Buckner, Andrews‐Hanna, & Schacter, 2008; Raichle, 2015). It is generally speculated that the functional configurations assumed by the brain cortex are reflective of underlying anatomical connection (Honey et al., 2009; Passingham, Stephan, & Kotter, 2002). In a network‐based framework, we speculated that the reduced FA and FN between IPL.L and ANG.R might be the potential structural basis for the modifications of DMN functional connectivity in patients with glioma (Ghumman, Fortin, Noel‐Lamy, Cunnane, & Whittingstall, 2016; Greicius, Supekar, Menon, & Dougherty, 2009; Maesawa et al., 2015). The exact relationship between functional networks and structural networks in glioma patients remains to be elucidated.

## 5 LIMITATIONS

This study has several limitations worth noting. First, similar to many imaging studies (Maesawa et al., 2015; Zhang et al., 2016), the current study was limited by a small number sample size, which prevented grouping based on tumor characteristics and making further stricter corrections. The results could have minimized or exaggerated the differences between HCs and patients with glioma. More accurate grouping (e.g., a subgroup of patients with low‐grade IDH‐mutated glioma) could have provided more information about the difference of WM integrity from a pathophysiological perspective.

Second, we could only detect a generalized effect of the heterogeneous glioma when all the patients were put together in one group. To compensate for this, we subdivided the patients into three subgroup level (frontal, left, or right temporal lobe) to analyze the effect of tumor locations on specific WM tracts. Consequently, subjects with glioma in parietal and occipital lobe were not sufficient for subgroup analysis. Third, although we selected the subjects with no evidence of shift of the midline, the mass effect from the lesion might distort the surrounding anatomy, resulting in imprecise parcellation. Deterministic or probabilistic tractography with individualized seeds is needed in the future studies to better delineate those subject‐specific tracts. Maier‐Hein et al. have previously put forward that there are still challenges in mapping the human connectome based on diffusion tractography (Maier‐Hein et al., 2017). We should bear in mind that the results of biomedical image analysis sometimes should be interpreted with care (Maier‐Hein et al., 2018). Considering the fundamental ambiguities inherent in tract reconstruction, therefore, there might exist invalid WM tract output in our results and certain findings of this study still need further verification.

Last but not least, we have to recognize that the clinical significance of the WM alteration cannot be either absolutely excluded or summarized from the specific cognition we have evaluated in the present study. Further studies should integrate a larger sample size and more comprehensive cognitive domains that can help to validate the specificity and commonality of clinical manifestation of cognition in patients with glioma.

## 6 CONCLUSION

In summary, the findings of the present study demonstrated altered integrity of WM tracts in patients with glioma. Fibers in the right hemisphere (particularly UF.R) seem to be more susceptible to pathological changes caused by invasive tumors in the frontal or right temporal lobe. The altered linkages (FA, FL, and FN) suggested that the reduced integrity of WM tracts may damage brain structural networks in patients with glioma, which might be associated with impaired cognitive function.

## CONFLICT OF INTEREST

None declared.

## AUTHOR CONTRIBUTIONS

HL, JC, and XH conceived and designed the experiments. DL, JC, and XH preprocessed and analyzed MRI data. KY, CX, JH, and ZL contributed materials/analysis tools. DL, GH, JC, XH, YL, and YZ involved in the preparation of the article, figures, and tables. DL, YL, and JC revised the manuscript. All authors read, revised, and approved the final version of the manuscript.

## Supporting information

Table S1‐S3

## Data Availability

The data that support the findings of the current study are available from the corresponding author upon reasonable request.
