# Supplementary material for: Alterations of white matter integrity associated with cognitive deficits in patients with glioma
Source: Brain Behav. 2020 May 15;10(7):e01639. doi: 10.1002/brb3.1639 (PMC7375068; doi:10.1002/brb3.1639)
Supplement: Supplementary file 1 — Table S1‐S3 [file BRB3-10-e01639-s001.docx]

| No. | Sex | Age  (year) | Tumor side | Tumor location | Peritumoral edema | WHO Grade | Paresis/Speech  disturbance | Pathological diagnosis |
| --- | --- | --- | --- | --- | --- | --- | --- | --- |
| 1 | F | 58 | Right | Frontal | slight | 2 | no | Astrocytoma |
| 2 | F | 36 | Right | Frontal | no | 2 | no | Astrocytoma |
| 3 | M | 52 | Right | Temporal/ insula | no | 2 | no | Astrocytoma |
| 4 | M | 59 | Right | Frontal | slight | 3 | no | Anaplastic Oligodendroglioma |
| 5 | F | 70 | Left | Temporal | slight | 4 | yes | Glioblastoma |
| 6 | M | 35 | Right | Temporal | medium | 2 | no | Astrocytoma |
| 7 | F | 46 | Left | Frontal | slight | 2 | no | Astrocytoma |
| 8 | M | 60 | Right | Temporal | slight | 4 | no | Glioblastoma |
| 9 | M | 61 | Left | Temporal | no | 3 | no | Anaplastic Astrocytoma |
| 10 | F | 55 | Right | Temporal/ insula | slight | 2 | yes | Astrocytoma |
| 11 | M | 58 | Right | Frontal | slight | 4 | no | Glioblastoma |
| 12 | F | 46 | Left | Frontal | no | 3 | no | Anaplastic Astrocytoma |
| 13 | M | 58 | Left | Temporal | slight | 4 | no | Glioblastoma |
| 14 | M | 43 | Left | Frontal | medium | 4 | yes | Glioblastoma |
| 15 | M | 66 | Right | Temporal | no | 2 | no | Oligodendroglioma |
| 16 | M | 53 | Right | Temporal/ insula | no | 2 | no | Astrocytoma |
| 17 | F | 38 | Right | Temporal | no | 2 | no | Astrocytoma |
| 18 | F | 38 | Left | Frontal | slight | 1 | no | Astrocytoma |
| 19 | M | 60 | Left | Frontal | slight | 3 | yes | Anaplastic Oligodendroglioma |
| 20 | F | 57 | Left | Temporal | slight | 4 | no | Glioblastoma |
| 21 | M | 59 | Right | Parietal | slight | 4 | yes | Glioblastoma |
| 22 | F | 33 | Right | Frontal | slight | 2 | no | Astrocytoma |
| 23 | F | 22 | Right | Parietal | no | 3 | no | Anaplastic Oligodendroglioma |
| 24 | M | 53 | Left | Frontal | slight | 4 | no | Glioblastoma |
| 25 | M | 67 | Right | Temporal/ insula | medium | 3 | no | Anaplastic Astrocytoma |
| 26 | F | 67 | Left | Occipital | no | 1 | no | Oligodendroglioma |
| 27 | F | 43 | Right | Temporal | no | 1 | no | Ganglioglioma |
| 28 | F | 65 | Right | Temporal/Occipital | slight | 4 | no | Glioblastoma |
| 29 | M | 16 | Left | Parietal | no | 1 | no | Ganglioglioma |
| 30 | M | 47 | Left | Temporal/ insula | no | 3 | yes | Anaplastic Oligodendroglioma |
| 31 | M | 21 | Right | Temporal | no | 1 | no | Ganglioglioma |
| 32 | F | 45 | Left | Temporal/occipital | slight | 4 | no | Glioblastoma |
| 33 | M | 59 | Right | Parietal | no | 3 | no | Anaplastic Oligodendroglioma |
| 34 | M | 63 | Left | Parietal | no | 4 | yes | Glioblastoma |
| 35 | F | 27 | Right | Frontal | no | 2 | no | Astrocytoma |

Table. S1. The demographics and clinical data of the patients.

Table. S2. The abbreviations and 20 Johns Hopkins University (JHU) white matter tracts template used in atlas-based analysis.

| **Index** | **White Matter Tracts/ Regions** | **Abbreviation** |
| --- | --- | --- |
| 1,2 | Anterior Thalamic Radiation (L/R) | ATR. L/R |
| 3,4 | Corticospinal Tract (L/R) | CST. L/R |
| 5,6 | Cingulum (Cingulate gyrus) (L/R) | CCG. L/R |
| 7,8 | Cingulum (Hippocampus) (L/R) | CH. L/R |
| 9,10 | Forceps Major / Forceps Minor | FCPM / FCPm |
| 11,12 | Inferior Fronto-Occipital Fasciculus (L/R) | IFOF. L/R |
| 13,14 | Inferior Longitudinal Fasciculus (L/R) | ILF. L/R |
| 15,16 | Superior Longitudinal Fasciculus (L/R) | SLF. L/R |
| 17,18 | Uncinate Fasciculus (L/R) | UF. L/R |
| 19,20 | SLF Temporal part (L/R) | SLFTP. L/R |

Table. S3. The abbreviations and regions of AAL template used in ROI-based network analysis.

| **Index** | **Regions** | **Abbreviation** |
| --- | --- | --- |
| 1,2 | Precentral gyrus | PreCG.L/R |
| 3,4 | Superior frontal gyrus (dorsal) | SFGdor.L/R |
| 5,6 | Orbitofrontal cortex (superior) | ORBsup.L/R |
| 7,8 | Middle frontal gyrus | MFG.L/R |
| 9,10 | Orbitofrontal cortex (middle) | ORBmid.L/R |
| 11,12 | Inferior frontal gyrus (opercular) | IFGoperc.L/R |
| 13,14 | Inferior frontal gyrus (triangular) | IFGtriang.L/R |
| 15,16 | Orbitofrontal cortex (inferior) | ORBinf.L/R |
| 17,18 | Rolandic operculum | ROL.L/R |
| 19,20 | Supplementary motor area | SMA.L/R |
| 21,22 | Olfactory | OLF.L/R |
| 23,24 | Superior frontal gyrus (medial) | SFGmed.L/R |
| 25,26 | Orbitofrontal cortex (medial) | ORBmed.L/R |
| 27,28 | Rectus gyrus | REC.L/R |
| 29,30 | Insula | INS.L/R |
| 31,32 | Anterior cingulate gyrus | ACG.L/R |
| 33,34 | Middle cingulate gyrus | MCG.L/R |
| 35,36 | Posterior cingulate gyrus | PCG.L/R |
| 37,38 | Hippocampus | HIP.L/R |
| 39,40 | Parahippocampal gyrus | PHG.L/R |
| 41,42 | Amygdala | AMYG.L/R |
| 43,44 | Calcarine | CAL.L/R |
| 45,46 | Cuneus | CUN.L/R |
| 47,48 | Lingual gyrus | LING.L/R |
| 49,50 | Superior occipital gyrus | SOG.L/R |
| 51,52 | Middle occipital gyrus | MOG.L/R |
| 53,54 | Inferior occipital gyrus | IOG.L/R |
| 55,56 | Fusiform gyrus | FFG.L/R |
| 57,58 | Postcentral gyrus | PoCG.L/R |
| 59,60 | Superior parietal gyrus | SPG.L/R |
| 61,62 | Inferior parietal lobule | IPL.L/R |
| 63,64 | Supramarginal gyrus | SMG.L/R |
| 65,66 | Angular gyrus | ANG.L/R |
| 67,68 | Precuneus | PCUN.L/R |
| 69,70 | Paracentral lobule | PCL.L/R |
| 71,72 | Caudate | CAU.L/R |
| 73,74 | Putamen | PUT.L/R |
| 75,76 | Pallidum | PAL.L/R |
| 77,78 | Talamus | THA.L/R |
| 79,80 | Heschl gyrus | HES.L/R |
| 81,82 | Superior temporal gyrus | STG.L/R |
| 83,84 | Temporal pole (superior) | TPOsup.L/R |
| 85,86 | Middle temporal gyrus | MTG.L/R |
| 87,88 | Temporal pole (middle) | TPOmid.L/R |
| 89,90 | Inferior temporal | ITG.L/R |
